# Abstracts of the 3^rd^ REDLARA Northwest Region Meeting, Quito, Ecuador, 8-9 November 2019

**DOI:** 10.5935/1518-0557.20190073

**Published:** 2020

**Table 1:** Results. Group 1, sperm selection by density gradient and group 2, sperm selection by Z-potential

Clinical Outcomes	Total (%)	Group 1 (%)	Group 2 (%)	*p*value
n of patients	290	149	141	-
n of mature oocytes	2641	1361	1280	-
Fertilization rate (%)	2081 (78.80)	1085 (79.7)	996 (77.81)	NS
Blastocyst development (%)	979 (47.04)	484 (44.60)	495 (49.70)	NS
n of transferred embryos	550	283	267	-
Positive *β*-hCG (%)	180 (62.06)	90 (60.40)	90 (63.83)	NS
Clinical pregnancy rate (%)	152 (52.41)	75 (50.34)	77 (54.61)	NS
Implantation rate (%)	206 (37.45)	97 (34.28)	109 (40.82)	NS
