# Supplementary material for: Abstracts of the 3rd REDLARA Northwest Region Meeting, Quito, Ecuador, 8-9 November 2019
Source: JBRA Assist Reprod. 2020 Jan-Mar;24(1):89. doi: 10.5935/1518-0557.20190073 (PMC6993158; doi:10.5935/1518-0557.20190073)
Supplement: Related texts

# Abstract: P1. Evaluation of pregnancy and implantation rates after microinjection of donated oocytes with sperm selected by Z-potential

**Authors:** Rocío Dávalos, Vannia Salazar, Belen Gramaglia, Alvaro Ascenzo, Milagros Revolledo, Alessandra Ascenzo, Fernando Peña

**Affiliations:** 1 Laboratorio de Reproducción Asistida, Clínica Miraflores, Lima, Peru.

**Keywords:** ICSI, Z-Potential, Implantation rate.

## Abstract

To evaluate whether the sperm selected by Z-potential improves the success rates of an assisted reproduction treatment with donated oocytes.

**Methods:**

The study was carried out in the Assisted Reproduction Laboratory of Clínica Miraflores during the period from June 2017 to December 2018. A total of 2641 human oocytes obtained from 290 patients undergoing ICSI treatment with donated oocytes were used. All cases were randomly divided into two groups: group 1, sperm selection by density gradient and group 2, sperm selection by Z-potential. All the zygotes (N=2081) obtained were cultivated for up to 5 or 6 days in K-Systems G185 incubators at 5.7% CO_2_ and 5% O_2_ concentration. The embryos that achieved the blastocyst stage were selected for embryo transfer.

**Results:**

Significant differences were not observed between the rates of fertilization, blastulation, clinical pregnancy and implantation.

**Conclusion:**

The results obtained in this study do not show statistically significant differences between both studied groups regarding all parameters studied. However, we observed a non-significant growing tendency in blastulation, implantation and clinical pregnancy rate. No improvement in the fertilization rate was observed.

# Abstract: P2. Towards to a single embryo transfer policy in a oocyte donation program

**Authors:** Luis Guzman, Naomi Inoue, Silvana Bardalez, Alicia Elias, Jazmin Meza, Wendy Montanchez, Javier Noriega-Portella, Abigail Oroche, Luis Noriega-Hoces, Luis Noriega-Portella

**Affiliations:** 1 PRANOR Laboratorio. Grupo de Reproducción Asistida. Lima, Peru.; 2 Clínica Concebir. Lima, Peru.

## Abstract

In oocyte donation programs, maternal age is generally above 40 years, this group of patients has higher incidence of perinatal and obstetric complication due to advance maternal age. Those complications become more complex when multiple pregnancies are achieved. Single embryo transfer (SET) could avoid risks associated with carrying multiple pregnancy such us preterm baby born, low birth weight and experience more adverse health outcomes than singleton infants. The aim of this study was to stablish a robust oocyte donation program with a single embryo transfer policy.

**Methods:**

A total of 2389 OD cycles were performed from January 2013 to December 2018. However, based on the aim of our study 870 OD cycles with fresh ET were included. The exclusion criteria were OD cycles plus PGT-A and total cryopreservation. Young women were selected as a donor's followed by strict selection criteria: genetic (karyotype and some donors were tested with carrier map), serological and psychological test. Donors were stimulated with GnRH antagonist protocol, using either hp-HMG (Menopur-Ferring^®^) or recombinant FSH (Gonal-Merck^®^) and GnRH antagonist (Cetrotide^®^), and triggered with Triptorelin 0.2 mg (Gonapeptyl^®^) when at least one follicle reached 18 mm. Oocyte donation was done either with fresh or vitrified oocytes.

**Results:**

Maternal age was 41.6±3.9, the overall clinical pregnancy rate (CPR) achieved 62% (542/870). CPR in SET and double embryo transfer (DET) was 53% (131/245) and 66% (409/617), respectively. Higher multiple pregnancy rate was observed in DET compared to SET (39% *vs.* 2%, *p*<0.001). When data was divided by years, the proportion of SET increased significantly in the last two years. Nevertheless, the preferential election for SET does not impact negatively our clinical pregnancy rates (Table 1).

**Conclusion:**

Oocyte donation program with SET policy followed by strict embryo selection tools decreased multiple pregnancy while no significant variation in clinical outcomes were observed.

**Table:** 

	Overall Clinical Pregnancy Rate	Percentage of Embryo Transfer with SET policy	Clinical Pregnancy in SET	Clinical Pregnancy in DET
**2013**	62%	16%a	42%	63%
**2014**	63%	23%a	45%	63%
**2015**	61%	17%a	40%	64%
**2016**	64%	22%a	54%	66%
**2017**	65%	40%b	52%	72%
**2018**	61%	43%b	53%	68%

^a,b^ (*p*<0.05)

# Abstract: P3. High clinical outcomes after deferred euploid embryo transfer in comparison with Freeze all policy and Fresh embryo transfer

**Authors:** Naomi Inoue, Carlos Zuñiga, Enrique Noriega, Maria Jose Mendiola, Pilar Pino, Silvana Marquez, Luis Noriega-Hoces, Luis Noriega-Portella, Luis Guzman

**Affiliations:** 1 PRANOR Laboratorio. Grupo de Reproducción Asistida. Lima-Peru; 2 Clínica Concebir. Lima, Peru.; 3 ADN diagnóstico. Lima, Peru.

## Abstract

To provide a critical assessment to improve clinical outcomes after the first embryo transfer determine by three different transfer policies in the same clinical laboratory.

**Methods:**

This prospective non-randomized study included 408 autologous oocyte cycles from January 2014 to July 2016, divided in three different groups according to the embryo transfer policy: fresh embryo transfer (ET), freeze all policy followed by a frozen-thawed embryo transfer (FA) and FA plus PGT-A.

**Results:**

Implantation rate achieved significantly higher levels in the FA plus PGT-A in comparison with the Fresh ET and FA group (61%, 24% and 34%, respectively). Additionally, clinical pregnancy rates (66%) were significantly higher in the FA plus PGT-A group compared to 37% and 45% in FRESH ET and FA respectively. In single embryo transfer, statistically increase pregnancy rates were also observed in the FA plus PGT-A group and the same tendency was observed when a good or fair quality embryo was transferred.

**Conclusion:**

Our findings suggest that the Freeze-all strategy in combination with PGT-A might improve clinical outcomes per embryo transfer and highlight the importance of a superior endometrium preparation in frozen-thawed embryo transfer.

# Abstract: P4.Oocyte competence is affected by Body Mass Index in women undergoing In Vitro Fertilization procedures

**Authors:** Javier García-Ferreyra, Jorge Carpio, Milton Zambrano, Pedro Valdivieso-Mejía, Pedro Valdivieso-Rivera

**Affiliations:** 1 Laboratory of Assisted Reproduction. Alcívar Hospital, Guayaquil, Ecuador.

## Abstract

At present, obesity is prevalent around the world, and incurs a public health problem involving ART patients. Women with high Body Mass Index (BMI) have several reproductive problems. However, the effect of BMI on oocyte competence, associated to oocyte reproductive function, is unclear. The objective of this study was to evaluate the effects of BMI on oocyte competence measured through developmental and embryonic qualities, and its capacity to produce gestational sacs.

**Methods:**

A total of 1265 oocytes that were obtained from 174 IVF/ICSI non-donor cycles and performed between January 2017 and December 2018 were analyzed. The oocytes were allocated according to BMI of patients in three groups: Normal group: 18.5-24.9 (*n*=403 oocytes), Overweight group: 25.0-29.9 (*n=*593 oocytes) and Obese group: ≥30.0 (*n=*269 oocytes). We compared fertilization rates, quality and cell numbers in embryos at day 3, development and quality of blastocyst, transferred embryos, and number of gestational sacs.

**Results:**

There was no difference in the seminal parameters and hormonal stimulation days in the study groups. Patients in both overweight and obese groups used more hormones compared to those of normal weight (*p*<0.05). Fertilization rate, percentages of cleaved zygotes, good-quality embryos at Day 3 and good blastocysts were similar in all study groups. The overweight and obese groups had embryos at Day 3 with less cells, and significantly less blastocyst development rate compared to group with normal BMI (*p*<0.05). Similar embryo numbers were transferred in the three evaluated groups, however, those with overweight and obesity showed significantly lower implantation rates compared to the group with normal BMI and they needed significantly more oocytes to obtain a successful gestational sac (*p*<0.05). Moreover, the obesity group needed a higher number of oocytes compared to the overweight group (*p*<0.05).

**Conclusion:**

An increase in BMI affects significantly oocyte competence, causing a minor blastocyst development and significantly reduced implantation rate. Patients with overweight and obesity needed significantly more oocytes to obtain a success gestational sac. Previous changes in diet, combined with weight loss activities, are highly recommended in patients that will undergo in vitro fertilization to improve their possibility of a successful pregnancy.

**Table 1:** Characteristics of patients according to their BMI

	Normal	Overweight	Obesity
Patients	57	82	35
BMI (Mean ± SD)	22.6±1.57	27.3±1.37 *	33.1±3.01 *, **
Female age (years) (Mean ± SD)	33.9±3.34	33.8±3.79	33.3±3.55
Stimulation days (Mean ± SD)	8.9±1.09	9.0±1.26	9.1±1.05
rFSH (U/L) (Mean ± SD)	1683.5±451.08	1902.1±396.39 *	2175.1±363.64 *, **

* *p*<0.05 in relation to normal group

** *p*<0.05 in relation to overweight group

**Table 2:** Comparison of laboratory results between the study groups

BMI	Normal	Overweight	Obesity
Oocytes	403	593	269
Fertilized oocytes (%)	353 (87.6)	506 (85.3)	228 (84.8)
Cleaved embryo Day 3 (%)	343 (97.2)	504 (99.6)	225 (98.7)
No. cell/embryo Day 3 (Mean ± SD)	7.3±0.75	7.0±0.81 *	6.9±1.04 *
Good-quality embryos Day 3 (%)	83.7	84.9	79.1
Blastocyst formation rate (%)	47.8	42.9 *	42.7 *
Good-quality blastocysts (%)	90.2	88.9	90.6
Embryo transferred (Mean ± SD)	104 (1.86±0.38)	155 (1.89±0.31)	66 (1.89±0.32)
Gestational sacs	59	56	19
Implantation rate (%)	56.7	36.1 *	28.8 *, **
No. oocytes / gestational sac	6.8	10.6 *	14.2 *, **
Miscarriage (%)	10.2	8.9	10.5

* *p*<0.05 in relation to normal group

** *p*<0.05 in relation to overweight group

# Abstract: P5. Sperm concentration, motility and DNA fragmentation dynamics after Z potential selection

**Authors:** Carlos Vivar Mateo, Víctor Nuñez Silvestre, Carlos Duarte Santa María

**Affiliations:** 1 Andrology Laboratory, Niu Vida, Specialized Center for Assisted Reproduction. Lima, Peru.

## Abstract

The aim of this study was to evaluate the sperm concentration, motility and fragmentation dynamics after performing the z potential technique for sperm selection. We focused on these parameters due to the importance they have in order to determine what type of technique to use in an assisted reproduction procedure.

**Methods:**

This is a prospective study that was carried out at Niu Vida, Assisted Reproduction Center (Lima, Peru). We received the approval of the Center's Ethics Committee. Seventy-four sperm samples were included from patients who came for fertility consultation and agreed to participate in this study. The parameters analyzed were sperm concentration, sperm motility, and sperm DNA fragmentation. At the beginning, an aliquot of 200 µl was separated from de fresh sperm sample (FS) to measure initial parameters. Then, samples were separated by density gradients technique (DGT), at the end of the washing phase a 200 µl aliquot was separated to measure post density gradients parameters. Finally, Z Potential was performed according to Duarte *et al.*, at the end, a 200 µl aliquot was separated to measure parameters post selection by Z Potential technique (PZT). DNA Fragmentation Index of each sample was measured by Sperm Chromatic Dispersion (SCD) technique using the Halosperm Commercial Kit (Spain).

**Results:**

We analyzed whether there were differences between the values ​​of the parameters evaluated after carrying out the sperm selection by density gradients compared to the parameters shown post separation with the potential Z technique. Nested ANOVA variance test was used to estimate the difference between the subgroup averages.

**Conclusions:**

Sperms can differ in their ability to fertilize oocytes in vitro according to the viability they achieved during the selection procedures. Z potential technique is well known to discriminate sperms with a low fragmentation rate, which is important for embryo development in vitro. It is important to have a pool of sperms with a good motility rate even if sperm concentration decreases after sperm selection. We demonstrate that sperm z potential selection does not significantly affect the sperm motility parameter compared to conventional density gradients technique (*p*>0.5) despite obtaining a lower sperm concentration rate (*p*<0.00001). Therefore, depending mainly on the final sperm concentration, we could use the potential Z technique for different assisted reproduction procedures and not only in specific ICSI cases. This means that we could perform this selection for intrauterine insemination and conventional in vitro fertilization. We reinforce this conclusion based in reported previous studies that achieve great embryo cleavage rates and blastocyst formation performing conventional IVF with a 0.5x10^6^/ml range of sperm concentration.

**Table 1:** Descriptive values and standard deviations for the three analyzed parameters

PARAMETERS	FS	POST DGT	POST PZT
**SPERM CONCENTRATION**	79.5473±5.95	33.473±2.94	13.4189±1.411
**SPERM MOTILITY**	52.1892±2.33	94.6622±0.01	96.0676±1.1132
**SPERM FRAGMENTATION**	17.3378±0.003	7.2973±0.002	2.6892±0.001

# Abstract: P6. IVF-successful pregnancy in patient with Kallman's syndrome: a case report

**Authors:** Germán David Ospina Idárraga, Iván Darío Montes Suarez, Katherine Gisell Hernández Osorio, Diana Milena Díaz Corredor

**Affiliations:** 1 Instituto de Fertilidad Humana Inser. Bogotá, Colombia.

## Abstract

Kallman's syndrome (KS) is the most frequent type of hypogonadotropic hypogonadism with a global incidence of 1 in 50.000 women (Yu *et al*., 2012).

**Objective:**

To describe a case of a woman with KS, who achieved pregnancy with live newborn, using assisted reproductive technique.

**Case Presentation:**

A 31-year-old, with desire for pregnancy, assisted to InSer Bogota in 2011. KS was diagnosed as a teenager. FSH and LH levels close to 0 and phenotypic characters of KS. She received hormonal replacement therapy for the past 14 years until 29 years old. Hysterosalpingogram with normal uterine morphology and tubal patency. AMH 4,1 ng/mL, normal pituitary magnetic resonance, GH, TSH, PRL y ACTH were normal. Transvaginal ultrasonography with uterus of 3.5X2.2X3.1 cm, right ovarian volume 2.8 cc, left ovarian volume 1.8cc. Semen analysis: vol 4 cc, concentration: 111 million/mL, progressive motility: A+B: 73%, morphology: 2%. It was initiated management with estradiol valerate for 3 months until achieving an appropriate uterine volume and functional endometrium. Afterward, an IVF cycle without GnRH analogues, ovarian stimulation with hMG 150 UI and rFSH 150 UI per day for 14 days. Oocyte retrieval were 6 follicles of 18 - 20 mm of diameter, trilaminar endometrium of 8.5 mm; triggering with r-hCG 250 mcg and ultrasound-guided follicular aspiration, getting 4 metaphase II oocytes. Two 72 hours-embryos were transferred. Luteal phase support with intravaginal progesterone 800 mg per day and estradiol valerate 8 mg per day and acetylsalicylic acid 100 mg per day. Two weeks later β-hCG 433.7 mUI/mlL and ultrasonography control at 4 weeks with intrauterine gestational sac of 49 mm and embryo of 8.4 mm with embryo cardiac activity. Posteriorly, Normal pregnancy course, cesarean delivery at 38 weeks, male newborn, weight: 3600 gr, Apgar 9/10 - 10/10.

**Comments:**

Choosing and carrying out a fertility treatment in SK patients, represents a medical and clinical challenge, considering pathophysiology's complexity, multiple clinical features and poor scientific evidence available. Pregnancy and live newborn rate depend on an adequate ovarian stimulation, using FSH and LH combinations (urinary or recombinant) and correct selection of ART (Gao *et al*., 2018). In high complexity techniques, it is not necessary to inhibit endogenous LH peak, due to base condition. We suggest adopting the protocol explained above, as a guide for the management of SK patients and pregnancy desire.

## References

[B1] Gao Y, Yu B, Mao J, Wang X, Nie M, Wu X (2018). Assisted reproductive techniques with congenital hypogonadotropic hypogonadism patients: a systematic review and meta-analysis. BMC Endocr Disord..

[B2] Yu HT, Lee CL, Huang HY, Soong YK (2012). Successful pregnancy in a woman with Kallmann's syndrome using human menopausal gonadotropin followed by low-dose human chorionic gonadotropin in the mid-to-late follicular phase. Taiwan J Obstet Gynecol.

# Abstract: P7. Prevalence of sperm fluid bacterial infections in patients evaluated at an Assisted Reproduction Center in Ecuador

**Authors:** Emilio Blum-Panchana, Gabriel Morey-León, Nicolás Gallardo-Molina, Xavier Blum-Rojas, Bernardo Blum-Pinto, Medardo Blum-Narváez, Tatiana Puga-Torres

**Affiliations:** 1 Santiago de Guayaquil Catholic University. Guayaquil, Ecuador.; 2 University of Guayaquil. Guayaquil, Ecuador.; 3 Medical School, Universidad Espíritu Santo (UEES). Samborondon, Ecuador.; 4 Innaifest Assisted Reproduction Center. Guayaquil, Ecuador.

## Abstract

The scarce symptomatology in patients with bacterial infections in the semen can give way to the lack of identification of pathogenic bacteria in men with fertility problems. Currently in Ecuador, there is a lack of studies on this issue. Therefore, the objective of this study is to determine the prevalence of pathogenic bacteria in the seminal fluid of patients with fertility problems treated at an assisted reproduction center in Ecuador.

**Methods:**

A retrospective analysis was performed to the spermogram results and semen bacteriological cultures of 426 patients who attended infertility consultation to undergo assisted reproduction treatment. The data was taken from patients who were treated between October 2018 and May 2019 at Innaifest assisted reproduction center. Semen profiles were evaluated according to WHO criteria, fifth edition. The seminal fluid pathogen evaluation was performed using the standard culture for gram positive and gram-negative bacteria. A statistical frequency analysis was performed. Data was also segmented by age range (20-29, 30-39, 40-49) for analysis.

**Results:**

The prevalence of bacterial infection in the seminal fluid of patients who attended a consultation to treat fertility problems was 13.38% (57 cases). The type of microorganism and distribution based on the age of the patients are described in tables 1 and 2, respectively. Among the patients with sperm quality alterations, a higher incidence of seminal fluid infection was found in cases with moderate (15.8%) and mild (17.5%) teratozoospermia.

**Conclusion:**

Our findings demonstrate a considerable incidence of bacterial infection in the seminal fluid of men attending fertility consultation, which demonstrate the importance of evaluation and treatment of it as part of the proper management of patients before starting an assisted reproduction procedure, within an assisted reproduction program.

**Table 1:** Bacteria identified in the culture

	Name	Percentage
Gram Negative Bacilli	*Escherichia coli*	49.10%
	*Proteus mirabilis*	24.60%
	*Proteus vulgaris*	1.80%
Gram-Positive cocci	*Staphylococus saprophyticus*	12.30%
	*Staphylococus epidermidis*	8.80%
	*Staphylococus aureus*	3.50%

**Table 2:** Distribution of the bacterial type based on the patient's age

	Patient Age Ranges		
Bacterial type	20-29 years	30-39 years	40-49 years
*Escherichia coli*	4	20	4
*Proteus mirabilis*	0	11	3
*Proteus vulgaris*	0	1	0
*Staphylococus saprophyticus*	0	6	1
*Staphylococus epidermidis*	0	3	2
*Staphylococus aureus*	0	2	0

# Abstract: P8. Low prognosis patients following the POSEIDON criteria: Evaluation of IVF cycles and outcomes in a University Reproductive Medicine Center

**Authors:** Eduardo Correa, Karina Sequeira, Ana Godoy, Pablo Céspedes

**Affiliations:** 1 Maternal and Child Research Institute (IDIMI), Faculty of Medicine, University of Chile. Santiago, Chile.; 2 San Borja Arriarán Clinical Hospital, University of Chile. Santiago, Chile.

## Abstract

To characterize low prognosis women of IVF cycles following Poseidon criteria in our population, studying their frequency and comparing their outcomes to controlled ovarian stimulation (COS) protocols.

**Methods:**

Observational, retrospective study on fresh intracytoplasmic sperm injection (ICSI) cycles performed at the Institute for Maternal and Child Research (IDIMI), San Borja-Arriaran Clinical Hospital, University of Chile, from January 1^st^ 2016 to December 31^st^ 2018. The study included all low prognosis patients according to Poseidon stratification: women younger and older than 35 years with adequate ovarian reserve parameters (AFC >5; AMH > 1.2 ng/ml) with less than 9 oocytes retrieved (groups 1 and 2; G1-2); and women younger and older than 35 years with poor ovarian reserve (AFC <5; AMH < 1.2 ng/ml; groups 3 and 4; G3-4). Exclusion criteria considered frozen-thawed embryo transfer, egg donation, fertility preservation and suspended before retrieval cycles. COS protocols provided gonadotropin releasing-hormone (GnRH) antagonist, using FSH and LH according to clinical practice. Outcomes considered were ovarian reserve biomarkers, gonadotropins dose, duration of stimulation, follicular output rate (FORT), total and mature oocytes retrieved, embryos achieved, transferred embryos and biochemical pregnancy rates. Data analysis was performed with ANOVA test to compare means between more than 2 groups. Significance was defined as *p*<0.05. Data are presented as mean ± standard error of the mean, with 95% confidence intervals (tables not shown).

**Results:**

830 fresh IVF aspirated cycles were performed between January 1^st^ 2016 and December 31^st^ 2018, of which 51.3% (n=426) fulfilled Poseidon criteria. The distribution among groups was 25.6% for G1, 37.3% for G2, 10.1% for G3 and 21% for G4. As expected, age and ovarian reserve biomarkers were significatively different, both being two of the three criteria for Poseidon stratification. 58% were women older than 35 years (G2-4), while 42% were young patients (G1-3) (*p*<0.01). AMH and AFC was poor in G3-4, while in 63% of women was adequate (G1-2) (*p*<0.01). BMI didn't show any differences (*p*=0.7).FORT was 68% in both groups with adequate ovarian reserve (G1-2), while in G3-4 significantly increased to 79 and 96,9% (*p*<0.01). Low FORT in G1-2 reflects women with unexpected ovarian hypo-response given their adequate ovarian reserve. Although days of stimulation wasn't different among groups (11 days, *p*=0.21), younger patients with normal reserve (G1) received significant less LH and FSH, the latter increasing progressively from G1 to G4 (*p*<0.01). Considering the high proportion of hypo-responders and the low dose of gonadotropins used, early detection and tailored COS for these women could improve outcomes. The number of total oocytes (6.9; 6.3; 6.3; 5.2), mature oocytes retrieved (5.5; 5.2; 5.1; 4.0) and total embryos (2.6; 2.1; 1.9; 1.6) were significantly higher in G1, and progressively declined to Poseidon group 4 (*p*<0.01). This reflects the importance of age on predicting ART outcomes: G1 had the best performance even with the lowest FSH/LH dose used; and young patients with poor ovarian reserve (G3) showing better outcomes than older patients with similar reserve (G4). The mean number of transferred embryos (1.5) didn't show differences among groups (*p*=0.05). Biochemical pregnancy showed significant differences (*p*<0.01). Again, the best performance was in G1 with 36%, declining progressively to G4 (27.5, 21.2 and 21.1%).

**Conclusion:**

Poseidon criteria and FORT allows the stratification of different groups of low prognosis women, like hypo-responders, facilitating tailored management to achieve better outcomes while improving the understanding of this heterogeneous patients.

# Abstract: P9. Term delivery of a dicavitary twin pregnancy in a didelphys uterus after single frozen embryo transfer in each cavity: a case report

**Authors:** Walter Osorio-Ramírez, Susana Salazar-Lopez, Diana Gomez-Cortésc, Diana Quintero-Cordoba, Natalia Posada-Villa

**Affiliations:** 1 Department of Gynecologist, division of Reproductive Medicine, Instituto Antioqueño de Reproducción. InSer, Medellín, Colombia.; 2 Assisted Reproduction Techniques Laboratory, Instituto Antioqueño de Reproducción, InSer. Medellín, Colombia.

## Abstract

Case report of a successful twin pregnancy with term birth in a patient with a double uterus after dual single embryo transfer in each cavity.

**Case Presentation:**

Thirty nine year old woman who consulted at InSer Medellín, with primary infertility associated with severe male factor, advanced maternal age and didelphys uterus type III with partial vaginal septum and a previous failed fresh in-vitro fertilization (IVF) intracytoplasmic sperm injection (ICSI) cycle with cryopreserved testicular sperm (cTESE). Endometrial preparation with estradiol valerate and intravaginal micronized progesterone followed by cryopreserved single embryo transfer in each uterine horn after two-failed fresh embryo transfer cycles (ICSI with cryopreserved testicular sperm). Two healthy male babies were born at 37±3 weeks that weighed 3600 and 3345 grams, respectively. No obstetric or perinatal complications were reported.

**Comments:**

We performed a dual frozen single embryo transfer into each horn of a bicornuate uterus, after two failed fresh embryo transfers with ICSI-cTESE, in this patient. In the first fresh cycle, the patient had a spontaneous abortion after elective single embryo transfer (eSET) into the right uterine horn and two good quality day 6 blastocysts were cryopreserved. Despite counseled otherwise, the patient chose to undergo a second ovarian stimulation cycle with ICSI-cTESE with no embryo transfer due to absence of embryo development to the blastocyst stage. The patient finally consented to a dual frozen single embryo transfer into each uterine cavity resulting in an uneventful dicavitary term twin gestation and elective cesarean section at 37+3 weeks. To our knowledge, this is the third published case report describing a successful twin dicavitary pregnany after split embryo transfer in a didelphys uterus^1^,^2^. Due to the extreme rarity of the condition, the obstetric and perinatal outcome of twin pregnancies in women with uterine anomalies is limited to a few case reports^3^,^4^. None of the case reports published so far report any obstetric or perinatal complications. However, it is quite possible that only cases with good pregnancy outcome were reported, resulting in biased and overly optimistic results. Also, the lack of comparative studies evaluating the obstetric outcome of twin gestations in patients with and without uterine anomalies limits the risk assessment of adverse pregnancy complications, such as preterm birth, cesarean delivery rate and fetal growth restriction associated with didelphys uterus^5^. Finally all published cases of dicavitary twin gestations have been delivered by caesarean section and the obstetric medical team should be prepared to handle maternal or fetal complications in a timely manner.

## References

[1] Yang MJ, Tseng JY, Chen CY, Li HY (2015). Delivery of double singleton pregnancies in a woman with a double uterus, double cervix, and complete septate vagina. J Chin Med Assoc.

[2] Aruh I, Aslan D, Karaarslan F, Pinar T, Keles R, Demir N (2005). Twin pregnancy in the horns of a bicornuate uterus after in vitro fertilization and split embryo transfer: a case report. J Reprod Med.

[3] Fox NS, Roman AS, Saltzman DH, Klauser CK, Rebarber A (2014). Twin pregnancy in patients with a uterine anomaly. J Matern Neonatal Med.

[4] Sugaya S (2010). Twin pregnancy after in vitro fertilization in a woman with a unicornuate uterus. Clin Exp Obstet Gynecol.

[5] Suh CS, Jee BC, Ku SY, Kim SH, Choi YM, Kim JG, Moon SY (2005). Twin gestation induced by clomiphene citrate and bromocriptine in both horns of a bicornuate uterus. Fertil Steril.
